# Digitally prefabricated versus conventionally fabricated implant-supported full-arch provisional prosthesis: a retrospective cohort study

**DOI:** 10.1186/s12903-022-02376-y

**Published:** 2022-08-09

**Authors:** Chaoqun Chen, Haiyan Lai, Huiyong Zhu, Xinhua Gu

**Affiliations:** Department of Stomatology, The First Affiliated Hospital, College of Medicine, Zhejiang University, #79 Qingchun Road, Hangzhou, 310003 Zhejiang Province People’s Republic of China

**Keywords:** Digital prefabrication, Provisional prosthesis, Implant-supported full-arch rehabilitation, Immediate loading

## Abstract

**Background:**

To evaluate and compare the clinical outcomes of digitally prefabricated and conventionally fabricated implant-supported full-arch provisional prostheses.

**Methods:**

In this retrospective study, a total of 39 patients (22 males and 17 females) who underwent implant-supported full-arch rehabilitation using the All-on-4 concept with an immediate loading protocol were included: 20 patients treated with digitally prefabricated provisional prostheses were assigned into Group A, and 19 patients treated with conventionally fabricated provisional prostheses were assigned into Group B. Implant/provisional prosthesis survival rates and complications were reviewed. Marginal bone loss (MBL) was investigated by CBCT. Surgical time, restorative time, and total operative time were analyzed. Postoperative pain and swelling were evaluated with the visual analog scale (VAS). The oral health impact profile (OHIP) questionnaire was administered before and after surgery.

**Results:**

The implant/provisional prosthesis survival rate was 100%, and complications appeared with low frequency in both groups, while the mean MBL was 0.30 ± 0.29 mm in Group A and 0.31 ± 0.41 mm in Group B after 3~ 6 months (*P* > 0.05). The average restorative time in Group A (116.16 ± 16.61 min) was significantly shorter than that in Group B (242.11 ± 30.14 min) (*P* < 0.05). Patients in Group A showed lower pain/swelling VAS scores after surgery than Group B (*P* < 0.05). Low OHIP scores with high satisfaction with the overall effects were shown in both groups.

**Conclusion:**

Prefabricated prostheses reduced the prosthetic time and postoperative discomfort in patients whose immediate rehabilitation was based on the All-on-4 concept. This prefabrication technology may be a predictable alternative to improve the short-term clinical outcome of implant-supported full-arch provisional rehabilitation.

## Background

Fixed implant-supported prostheses with immediate loading protocols have become a normal practice when treating edentulous patients. An immediate prosthesis can meet the functional and esthetic needs of the patients throughout the treatment stage and can improve patients’ quality of life significantly [1–3]. After long-term follow-up, the immediate loading of implant-supported fixed prostheses proved to be a reliable technique [4–8]. Malò and colleagues presented the All-on-4 concept and reported high success and survival rates of the prostheses and implants [1, 2]. Recently, a systematic review showed the high oral health-related quality of life (OHRQoL) and satisfaction in patients whose rehabilitation was based on the All-on-4 concept with implant-supported full-arch prostheses [9].

Conventionally, the provisional restorative procedure after implant operations on edentulous patients is complex and time-consuming, as it includes impression taking, interocclusal recording, prosthesis fabrication and delivery. To obtain an immediate prosthesis, patients who complete only implant-placement surgery still have to suffer a lengthy and complicated restorative procedure, and the prolonged exposure of the surgical area may increase the risk of postoperative discomfort. Therefore, the conventional fabrication of implant-supported full-arch provisional prostheses may affect the postoperative satisfaction of such patients.

With advances in all aspects of digital techniques, precise preoperative planning for implant surgery and prefabricated implant-supported provisional prosthesis has become feasible [10]. Prefabricated prostheses can better achieve esthetic and functional outcomes at the time of surgery [11, 12]. Data obtained using cone-beam computerized tomography (CBCT) can be imported into implant planning software programs to analyze the surrounding vital anatomic structures to determine the ideal implant locations [13]. Intraoral scanning devices help create a more realistic view of the intraoral soft tissues [14]. Optimal prosthetic-driven implant placement can be scheduled virtually before surgery using a scanning template [15]. Digital data from CBCT and intraoral scans can be directly transferred to the manufacturer of surgical templates and provisional prostheses [16, 17].

At present, just a few studies have reported on digitally prefabricated provisional prostheses [17–19], and to our knowledge, the existing literature has not compared the clinical efficacy of digitally prefabricated and conventionally fabricated implant-supported full-arch provisional prostheses, especially regarding the difference in postoperative discomfort from the perspective of patients. Therefore, the aim of this study was to compare the short-term clinical outcomes of a digitally prefabricated implant-supported full-arch provisional prosthesis with those of a conventionally fabricated provisional prosthesis.

## Methods

### Study design and patient selection

This retrospective study was approved by the local University Hospital Research Ethics Board (No. IIT20220009B-R1), and the research procedure followed the Helsinki Declaration issue. The medical records of patients from The First Affiliated Hospital of Zhejiang University School of Medicine was reviewed. The study included patients who received immediately implant-supported full-arch restoration based on the All-on-4 concept with an immediate loading rehabilitation between June 2019 and December 2021. A total of 39 patients (22 males and 17 females, 16 partially and 23 completely edentulous jaws) were included in the study. The time since tooth extraction varied from the day of implant surgery to more than 10 years. Exclusion criteria were existing uncontrolled systemic disease, and heavy smoking (more than 20 cigarettes per day). All participants had signed the informed consent form prior to the treatment.

According to the type of provisional prosthesis, the patients were assigned to two groups. Twenty patients treated with digitally prefabricated provisional prostheses were assigned into Group A, and 19 patients treated with conventionally fabricated provisional prostheses were assigned into Group B. In both groups, template-guided implant surgeries were carried out.

### Preoperative procedures

**Fig. 1:**
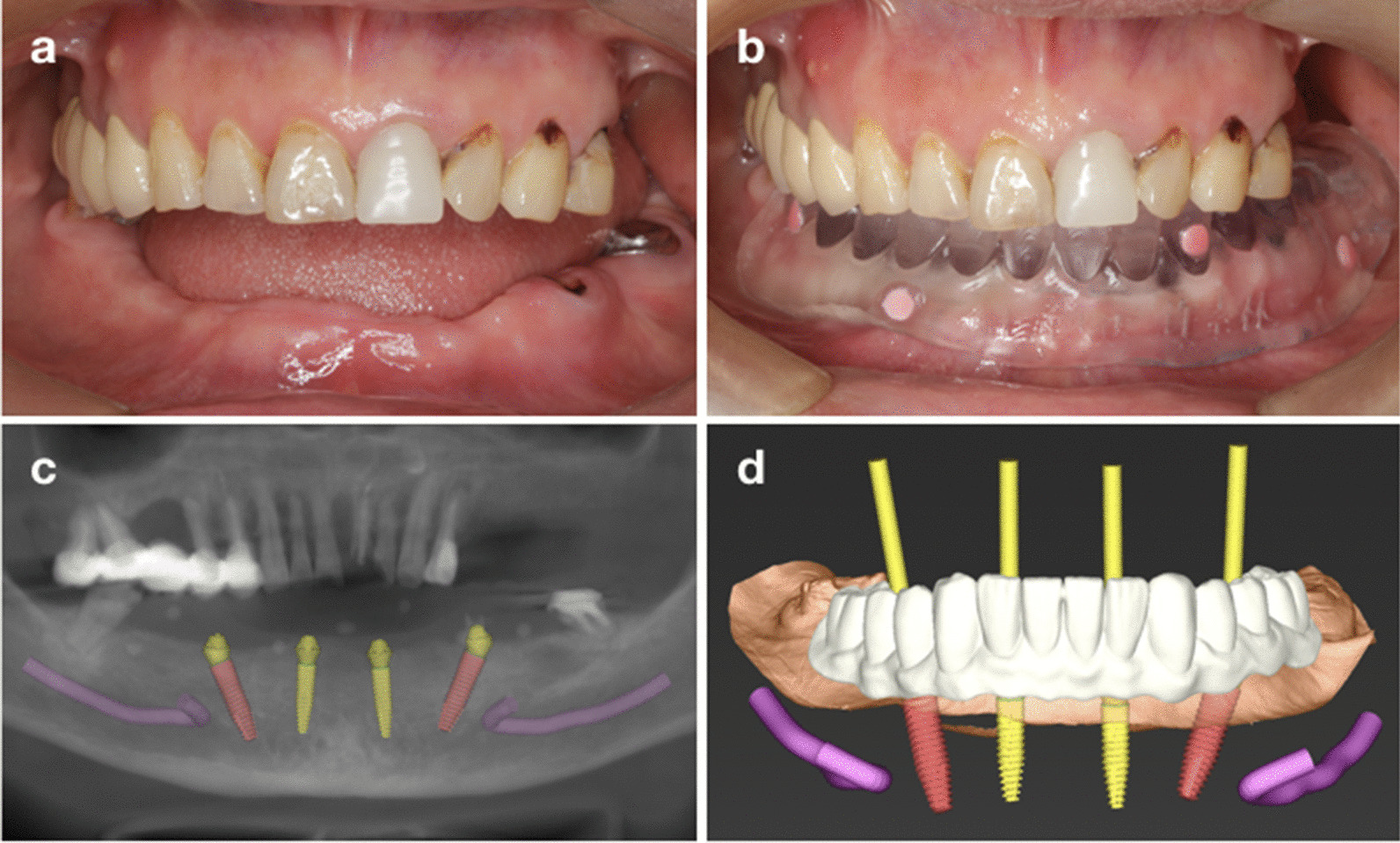
Preoperative procedure in Group A. **a** Intraoral examination; **b** radiographic template; **c** preoperative CBCT scan and planned implants; **d** digital design of the implants and prosthesis

**Fig. 2:**
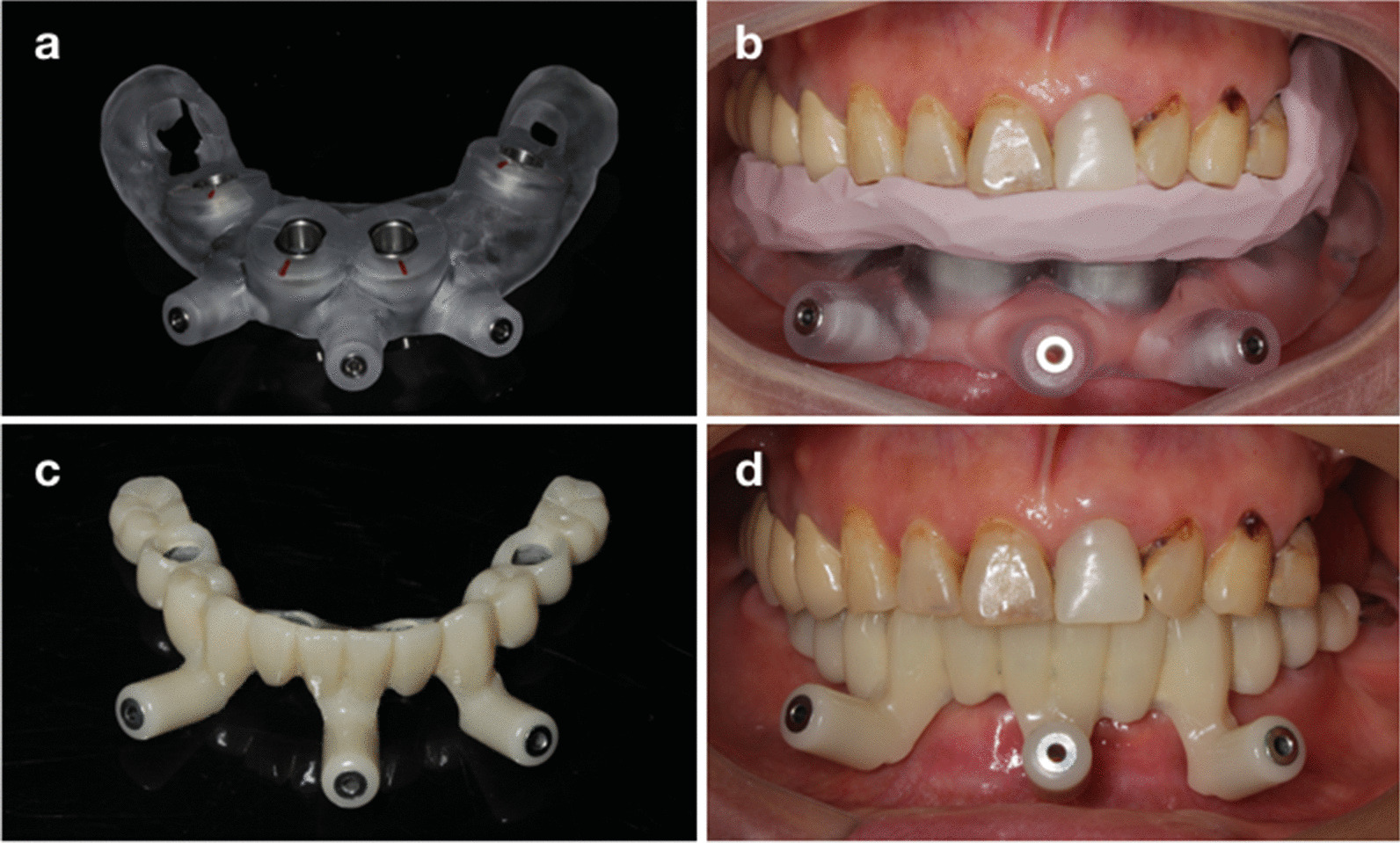
Computer-aided manufacturing of the surgical templates and provisional prosthesis. (**a**, **b**) Surgical templates; (**c**, **d**) Prefabricated provisional prosthesis

CBCT radiographs were taken to collect detailed three-dimensional information on the patients’ maxillofacial hard tissues. Patients’ preliminary impressions (Impregum™, 3 M ESPE) and intraoral scans was collected and used to fabricate a diagnostic cast and create a radiographic template. Then, a computer-assisted implant design was performed in a prosthetically oriented way (Fig. 1). Surgical templates (for patients in both groups) and provisional restorations (for patients in Group A) were created using a 3D printer (Fig. 2).

### Surgical intervention

Implant surgeries were performed by an experienced dentist (Xinhua Gu). A mini-flap approach was performed after local anesthesia (Primacaine Adrenaline; Pierre Rolland). Surgical templates were carefully fitted intraorally and stabilized with several fixation pins. Drilling of each implant site was accurately guided, 4 to 6 implants (Ankylos, Dentsply Sirona/ Straumann SLA, Institute Straumann AG/ Nobel Bioactive, Nobel Biocare) were immediately inserted in each jaw and a minimum insertion torque of 35 Ncm was obtained following the recommended protocol to obtain primary stability. Straight or angulated temporary abutments were screwed on top of the implants based on the preoperative digital design. The flap was repositioned and sutured (VICRYL Plus, Ethicon). CBCT was taken to evaluate the positions of the implants and abutments.

### Immediate provisional prosthesis

**Fig. 3:**
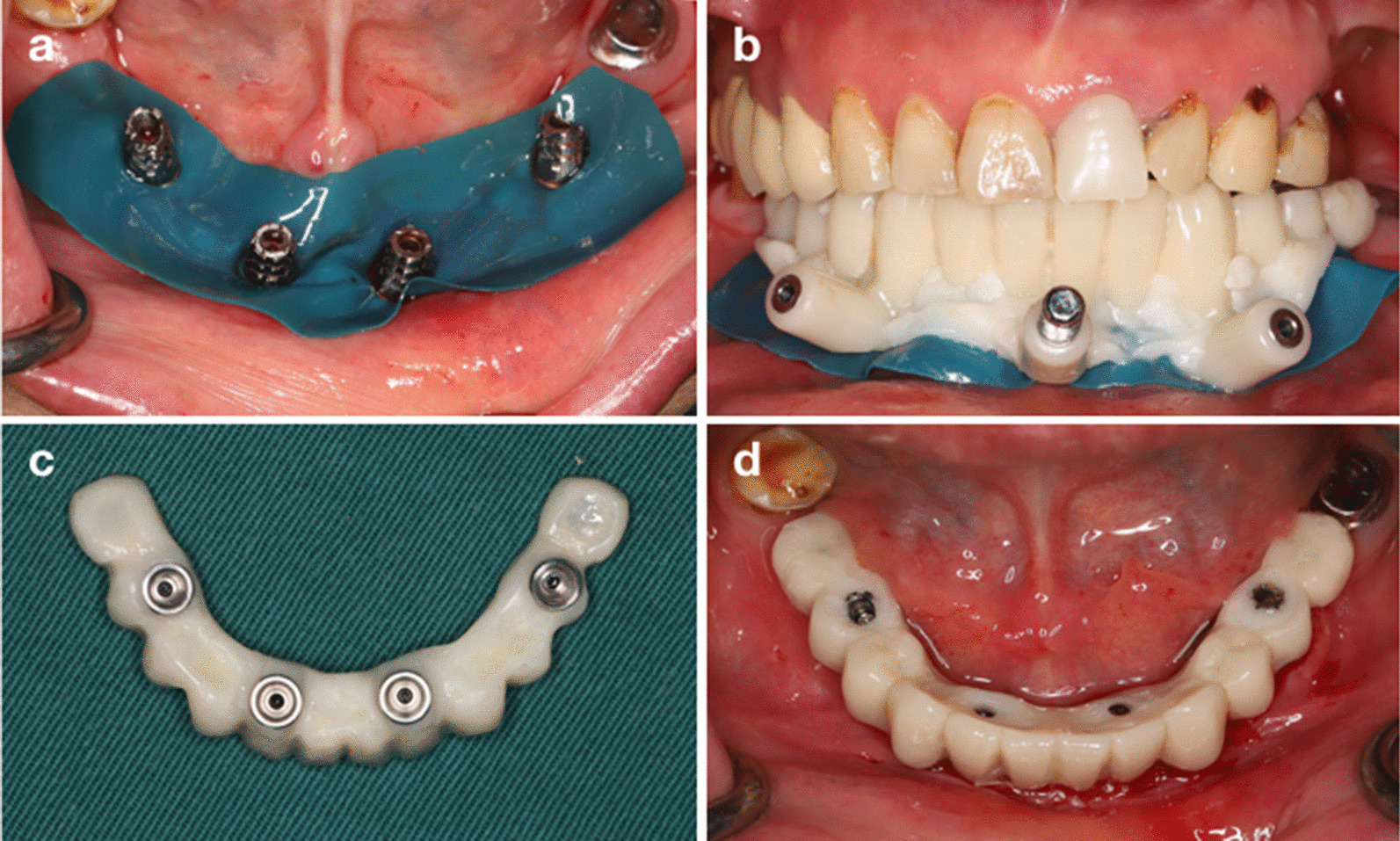
Provisional prosthetic protocol. **a** isolation of the surgical field; **b** passive fit of the provisional prosthesis; **c** further modification; **d** fixation of the provisional prosthesis

In Group A, a digitally prefabricated provisional prosthesis with a metal framework reinforced acrylic resin-based restoration was fixed to the implants immediately after surgery. First, the surgical field was isolated using a rubber dam. The provisional prosthesis was positioned and stabilized with pins. Minor modifications were made when necessary to ensure passive fit. Then, the provisional prosthesis was connected to titanium copings by auto-polymerizing acrylic resin (Luxatemp Star, DMG). After the acrylic resin polymerized, the prosthesis was removed from the oral cavity, further modified and polished, and then delivered to the patients after checking occlusion (Fig. 3).

In Group B, the conventional fabrication of a full-arch acrylic resin provisional prosthesis was conducted immediately after implant placement surgery. First, an impression was taken using silicone elastomeric material (Impregum™, 3 M ESPE) and interocclusal recording was performed. Then, a wax-up prosthesis was made and modified intraorally. After that, provisional full-arch acrylic resin dentures were manufactured and fixed to the implants. Finally, the occlusion was checked, and modifications were made if necessary [20].

**Fig. 4:**
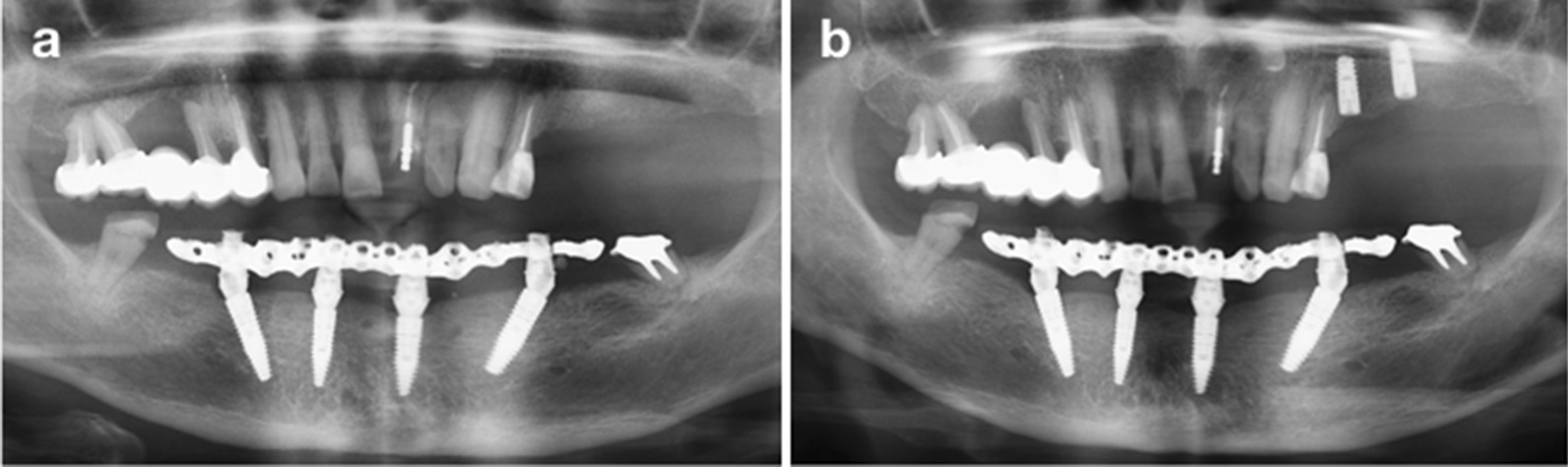
Radiographs. **a** Panoramic radiograph after implant surgery and immediate loading. **b** Panoramic radiograph at the 6-month follow-up (before definitive restoration): stable marginal bone levels were shown

Panoramic radiographs were obtained to verify the coupling between the prosthetic components and the secondary abutments (Fig. 4). The screws were tightened at the recommended torque, and the screw access holes were sealed with composite resin (Filtek ^TM^ Z350 XT, 3 M ESPE). The prosthetic time was recorded in both groups in this procedure.

### Postoperative maintenance

All the patients were prescribed cephalosporin (500 mg, twice daily), metronidazole (200 mg, three times daily), and ibuprofen (600 mg, three times daily, if needed) for three days after surgery. In addition, 0.2% chlorhexidine mouthwash was administered after meals for two weeks as postoperative care for all the participants. The patients were also instructed to clean their prostheses and the gap between their mucosa and the prostheses.

### Postoperative evaluation and follow-up

The total operative time, including the surgical time and restorative time, were recorded. Postoperative pain and swelling were evaluated using a visual analog scale (VAS) ten days postoperatively [21]. The marginal bone level (MBL) around the implant was measured from the CBCT scans immediately after surgery and then 3–6 months after surgery. Implant/provisional prosthesis survival and complications were recorded. Complications were categorized into biological, mechanical, and functional complications. Biological complications mainly included fistula and abscess formation and peri-implant pathology. Mechanical complications consisted of fracture or loosening of screws/abutments/prostheses and detachment of crowns from their denture base. Functional complications were identified as masticatory dysfunction, cheek/lip biting, articulation disorders, poor comfort, and poor hygiene [20]. Factors related to OHRQoL were assessed using the oral health impact profile (OHIP) questionnaire. The questionnaire was administered on three occasions: before surgery (T0), 10 days after prosthesis delivery (T1), and 3–6 months after surgery (T2). To avoid bias, participants completed the questionnaires independently in the absence of the researchers.

### Statistical analysis

All data analysis was performed using SPSS software (ver. 22.0; SPSS Inc., Chicago, IL, USA). Descriptive analysis results were presented as means, standard deviations (SD) and 95% confidence interval (CI). To determine the normal distribution of the measurements, a Kolmogorov-Smirnov test was performed. Depending on the distribution, a Student *t* test or Mann–Whitney U test was used to identify any significant differences. Furthermore, multivariate tests and Mauchly’s test of sphericity were performed to analyze the pain/swelling VAS and OHIP scores of patients. Proportions, means, SDs, medians, and 25th and 75th percentiles were used as summary statistics.

## Results

Thirty-nine patients (22 males and 17 females; average age: 56, ranging from 29 to 82) were included in the study (Table 1). A total of 200 implants were placed in 18 maxillae (46.15%) and 21 mandibles (53.85%). All the implants had achieved peak insertion torque. Table 1 lists the details of the included patients. All the patients received a 10~12-unit provisional prosthesis based on the All-on-4 concept on the day of surgery with an implant healing period of 3–6 months according to the patients’ individual conditions.

**Table 1:** Patient demographic data

Groups	Group A	Group B
Age (mean)	57.85	54.82
Gender (male/female)	12/8	10/9
Jaw (maxillary/mandibular)	8/12	10/9
Dental arches (partially/completely edentulous)	9/11	7/12
Antagonist (natural teeth/removable dentures)	13/7	11/8
Implant number (mean, SD)	5.1 (0.91)	5.16 (1.07)
Follow-up months (mean)	5.15	5.47

After 3–6 months’ follow-up, no implant or prosthesis failure was registered, indicating a 100% implant/provisional prosthesis survival rate in both groups. The mean MBL was 0.30 mm (SD: 0.29 mm) in Group A and 0.31 mm (SD: 0.41 mm) in Group B. According to the Kolmogorov-Smirnov test, the MBL of both groups conformed to a normal distribution (*P* = 0.200). T-test results showed no significant difference between the two groups (*P* = 0.897). During the follow-up period, no biological complications were recorded. Mechanical complications appeared with low frequency (Table 2).

**Table 2:** Distribution of postoperative complications

Complications	Group A Patient/occurrences	Group B Patient/occurrences
Mechanical complications		
Prosthetic fracture	0	0
Loose screw	1/2	2/3
Artificial tooth separation	1/1	1/1
Functional complications		
Phonetic problems	3	4
Biological complications	0	0

Surgical time, prosthetic time, and total operative time were analyzed. According to the Kolmogorov-Smirnov test, the surgical time (81.15 ± 15.01 in Group A, 82.26 ± 16.52 in Group B) conformed to a normal distribution (*P* = 0.200), while the prosthetic time (116.16 ± 16.61 in Group A, 242.11 ± 30.14 in Group B, *P* = 0.000) and total operation time (195.65 ± 26.09 in Group A, 324.37 ± 41.86 in Group B, *P* = 0.033) did not conform to a normal distribution. T-testing showed no significant difference between the two groups in surgical time (*P* = 0.827). The Mann–Whitney U test showed a significantly shorter prosthetic/total operation time in Group A than in Group B (*P* < 0.05).

**Fig. 5:**
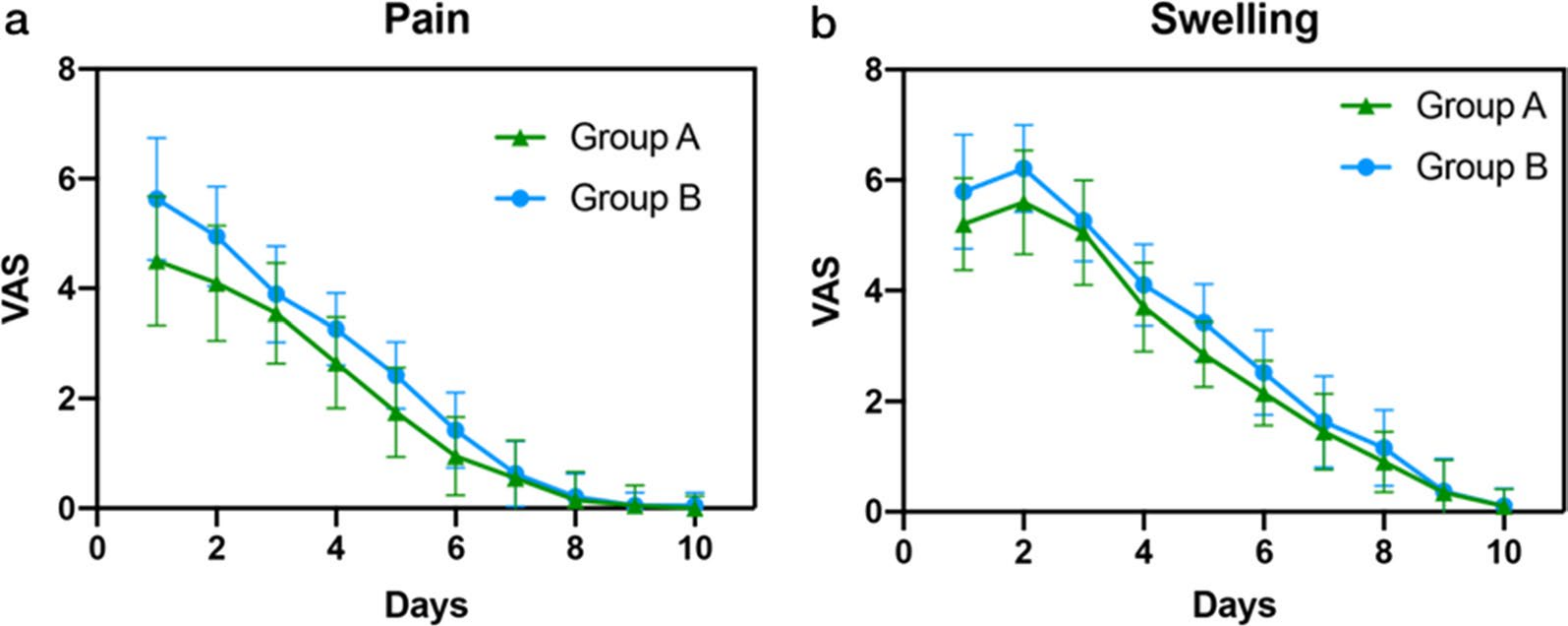
Postoperative discomfort. **a** Postoperative pain curves (pain-VAS) over a period of 10 days. **b** Postoperative swelling curves (swelling-VAS) over a period of 10 days

VAS scores are shown in Fig. 5. Multivariate analysis of the VAS scores at different measurement intervals showed significant differences in pain scores (*P* = 0.032) and swelling scores (P = 0.000). The pain severity on days 3 and 6 in Group B was significantly higher than that in Group A (*P* = 0.016). Postoperative swelling peaked on Day 2. Patients in Group B reported a swelling score of 6.2 on average, and those in Group A reported a score of 5.6 on average. No significant difference was found between two groups (*P* = 0.073).

OHIP scores are shown in Tables 3 and 4. Subscale scores revealed that functional limitations, physical pain, psychological discomfort and physical limitation domains had a major negative impact in both groups at T0. Physical limitation, physical pain, and psychological limitation scores decreased significantly with time in both groups. Median scores of physical pain and psychological limitation remained unchanged from T0 to T1. Multivariate analysis of OHIP scores at different time intervals showed significant differences between different time intervals (*P* < 0.05). The lowest OHIP score was found at T2 in all the subscales.

**Table 3:** Subscale scores of OHIP changes, median (25th-75th percentile)

Subscales	Q	Group A			Group B		
		T0	T1	T2	T0	T1	T2
Functional limitation	1	3 (2–3)	2 (1–2)	0 (0–0)	3 (2–3)	2 (1–2)	0 (0–0)
	2	3 (2–3)	1 (1–2)	0 (0–0)	3 (2–3)	1 (1–2)	0 (0–0)
Physical pain	3	2 (1-2.25)	2 (1.75–2.25)	0 (0–0)	2 (1–2)	2 (2–3)	0 (0–0)
	4	3 (2-3.25)	1 (0–2)	0 (0–0)	3 (2-3.5)	1 (1-1.5)	0 (0–0)
Psychological discomfort	5	2 (2–3)	1 (0–1)	0 (0–0)	2 (2–3)	1 (0.5-1)	0 (0–0)
	6	2 (1–2)	1 (1–1)	0 (0–0)	2 (1–2)	1 (0–1)	0 (0–0)
Physical limitation	7	3 (2-3.25)	1 (0.75-1)	0 (0–0)	3 (2–3)	1 (0-1.5)	0 (0–0)
	8	2 (1–2)	2 (1–2)	0 (0–0)	2 (1–2)	2 (1–2)	0 (0–0)
Psychological limitation	9	1.5 (0.75-2)	1 (1–2)	0 (0–0)	1 (0–2)	1 (1–2)	0 (0–0)
	10	2 (1–2)	0 (0–0)	0 (0–0)	2(1–2)	0 (0–0)	0 (0–0)
Social limitation	11	0 (0–0)	0 (0–0)	0 (0–0)	0 (0–0)	0 (0–0)	0 (0–0)
	12	1 (0–1)	0 (0–0)	0 (0–0)	1 (0–1)	0 (0–0)	0 (0–0)
Incapacity (handicap)	13	0 (0–1)	0 (0–0)	0 (0–0)	0 (0–0)	0 (0–0)	0 (0–0)
	14	0 (0–0)	0 (0–0)	0 (0–0)	0 (0–0)	0 (0–0)	0 (0–0)

**Table 4:** Statistical findings of summary scores of the OHIP, mean (SD) (95% CI)

Group	T0	T1	T2
Group A	23.15 (5.18)	11.8 (3.02)	0.25 (0.45)
Group B	21.53 (4.42)	12.53 (2.78)	0.47 (0.96)

Statistically significant differences were found between T0, T1, and T2 (*P* = 0.000).

No significant differences were found between Group A and Group B (*P* = 0.739).

## Discussion

This study aimed to compare digitally prefabricated with conventionally fabricated implant-supported full-arch provisional prostheses by evaluating the clinical outcomes (operation time, implant/prostheses survival rate, MBL, complications) and patients’ postoperative records (pain/swelling VAS and OHIP scores). Digital prefabrication of an implant-supported full-arch prosthesis is considered to be a predictable strategy for the immediate restoration of edentulous patients due to the shorter restorative/total operative time, lower postoperative VAS scores and high implant/provisional prosthesis survival rates.

Conventionally, the manufacturing of an immediate prosthesis requires the patient to cooperate with the dentist in taking the impression, creating an interocclusal recording, and waiting for a long time for the prosthesis to be fabricated. Patients generally suffer from intraoral bleeding and pain, and are in a state of fatigue after surgery. The time-consuming and complicated procedures may increase the risk of postoperative infections and make patients feel discomfort [22]. However, the digitally prefabricated prosthesis can take full advantage of digital technologies, which simplify the restorative procedure by eliminating several treatment steps [23]. A prospective pilot cohort study regarding computer-assisted full-arch immediate loading with digitally prefabricated provisional prostheses without casts has reported the advantage of digital impressions [17]. The objective of patient-oriented treatments includes minimally invasive surgery and low postoperative discomfort. The management of postoperative outcomes is important to improve patients’ postoperative experience and decrease their discomfort [22, 24]. There were lower pain scores in Group A, which was most likely attributed to the simplified immediate prefabricated restorative procedure. The prefabricated prosthesis requires only minor modifications, thereby decreasing the operation time, bleeding, and inflammation.

Despite the benefits of the prefabricated prosthetic protocol, precise passive fit of the prosthesis may be challenging due to the discrepancies that are involved in all indirect technical and clinical steps [25]. Successful prefabricated provisional reconstruction depends on accurate design and accurate implant insertion [13]. When a prefabricated prosthesis is intended to be used for a screw-retained acrylic resin prosthesis, great attention should be paid to the improvement of accuracy. A recent systematic review involving more than 1,400 implants revealed a total mean deviation of 1.12 mm at the implant entry point and 1.39 mm at the apex [26]. Since error was unavoidable, the sleeve hole diameter of the provisional prosthesis was designed to be 1.5 mm larger than the diameter of the secondary abutment to ensure passive fit.

In the present study, the cumulative implant survival rate was 100% with a follow-up of 3–6 months, comparable to other reports on immediate/early loading protocols and delayed loading protocols [1, 2, 27, 28]. Mechanical complications of resin tooth fracture occurred in two patients, including a maxillary canine in Group A and a mandibular incisor in Group B. Clinically, there is no need to replace the prosthesis when resin tooth fracture happens, indicating a 100% prosthetic survival rate in both groups. No correlation was found between resin tooth fractures and prosthesis type. Occlusal overloading caused by oral parafunctional activities such as bruxism is the major etiologic factor in the biomechanical complications of implant treatment [29]. Therefore, a careful occlusal adjustment should be carried out to acquire better stress distribution and help establish functional contacts to avoid stresses caused by oblique forces during eccentric movements in both groups [30].

In our study, because the number of implants varied from 4 to 6 and some patients needed tooth extraction during surgery, the scope of surgery was discrepant, which may have affected the postoperative outcomes. However, a current systematic review indicated no relationship of the number of implants used to support a complete-arch prosthesis with implant survival rate, prosthesis survival rate, prosthesis complications, or marginal bone loss in studies with follow-up periods between 5 and 15 years [31]. So far, immediate loading protocols for the maxilla is regarded to be a factor for limited success rates due to the different bone quality, which is more trabecular and softer in nature compared to that of the mandible [25, 27]. However, the implant survival rates between maxillae and mandibles in our study did not yield significant differences, which is in accordance with the results reported by Robert Niedermaier and colleagues [27].

High patient satisfaction is the major advantage of immediate loading, particularly during the early healing stage [32]. In the present study, high patient satisfaction was achieved once their prosthesis was in place. The overall OHIP scores were decreased significantly after provisional prostheses in both groups, reflecting improved oral-health-related quality of life after provisional reconstruction.

This study still has some limitations. First, it was a single-center study with only a limited number of participants. Second, some of the outcome indicators were subjective, and patient diversity might have impacted the results. Therefore, further well-designed multi-center randomized clinical trials with long-term follow-ups are necessary to confirm the results of this study.

## Conclusion

Considering the outcomes of postoperative pain/swelling, implant/provisional prosthesis survival, complications, MBL, and patients’ subjective evaluation with 3–6 months of follow-up, the digital prefabrication technique involving an implant-supported full-arch provisional prosthesis might be a viable treatment option in edentulous patients due to the simplified restorative procedure and improved satisfaction. However, further well-designed and long-term clinical trials are required to validate its use in implant dentistry. In addition, further optimization is necessary to improve the accuracy of the prefabricated prosthetic protocol.

## Data Availability

The datasets used and/or analysed during the current study are available from the corresponding author on reasonable request.

## Abbreviations

MBL: Marginal bone loss
CBCT: Cone-beam computed tomography
VAS: Visual analog scale.
OHIP: Oral health impact profile
